# Pegivirus avoids immune recognition but does not attenuate acute-phase disease in a macaque model of HIV infection

**DOI:** 10.1371/journal.ppat.1006692

**Published:** 2017-10-26

**Authors:** Adam L. Bailey, Connor R. Buechler, Daniel R. Matson, Eric J. Peterson, Kevin G. Brunner, Mariel S. Mohns, Meghan Breitbach, Laurel M. Stewart, Adam J. Ericsen, Christina M. Newman, Michelle R. Koenig, Emma Mohr, John Tan, Saverio Capuano, Heather A. Simmons, David T. Yang, David H. O’Connor

**Affiliations:** 1 Wisconsin National Primate Research Center, Madison, Wisconsin, United States of America; 2 Department of Pathology and Laboratory Medicine, University of Wisconsin–Madison, Madison, Wisconsin, United States of America; 3 Roche Sequencing Solutions, Madison, Wisconsin, United States of America; Emory University, UNITED STATES

## Abstract

Human pegivirus (HPgV) protects HIV+ people from HIV-associated disease, but the mechanism of this protective effect remains poorly understood. We sequentially infected cynomolgus macaques with simian pegivirus (SPgV) and simian immunodeficiency virus (SIV) to model HIV+HPgV co-infection. SPgV had no effect on acute-phase SIV pathogenesis–as measured by SIV viral load, CD4+ T cell destruction, immune activation, or adaptive immune responses–suggesting that HPgV’s protective effect is exerted primarily during the chronic phase of HIV infection. We also examined the immune response to SPgV in unprecedented detail, and found that this virus elicits virtually no activation of the immune system despite persistently high titers in the blood over long periods of time. Overall, this study expands our understanding of the pegiviruses–an understudied group of viruses with a high prevalence in the global human population–and suggests that the protective effect observed in HIV+HPgV co-infected people occurs primarily during the chronic phase of HIV infection.

## Author summary

People infected with HIV live longer, healthier lives when they are co-infected with the human pegivirus (HPgV)–an understudied virus with a high prevalence in the global human population. To better understand how HPgV protects people with HIV from HIV-associated disease, we infected macaques with simian versions of these two viruses (SPgV and SIV). We found that SPgV had no impact on SIV-associated disease early during the course of SIV infection–a time when SIV and HIV are known to cause irreversible damage to the immune system. Oddly, we found that the immune system did not recognize SPgV; a finding that warrants further investigation. Overall, this study greatly expands on our understanding of the pegiviruses and their interaction with the immune system.

## Introduction

Human pegivirus (HPgV)–formerly known as GB virus C (GBV-C) and also as Hepatitis G Virus (HGV)–is a positive-sense, single-stranded RNA virus in the Pegivirus genus of the *Flaviviridae* family [1]. HPgV infects one out of six humans globally and is frequently transmitted via blood products [2]. Little is known about the molecular biology of pegiviruses and the natural course of HPgV infection is poorly understood. However, HPgV causes persistent, high-titer viremia without eliciting symptoms or overt signs of disease [3,4]. Interestingly, epidemiological studies have found that people infected with human immunodeficiency virus (HIV) experience reduced disease when they are co-infected with HPgV. Specifically, HIV-infected individuals co-infected with HPgV are protected from HIV-induced CD4 T cell depletion [5–8] and pathological immune activation [9–12]. These individuals also experience a 2.5-fold reduction in all-cause mortality relative to HIV+ individuals not co-infected with HPgV (see [13] for a meta-analysis and [2] for a review). However, the timing and mechanistic underpinnings of this protective association are not known, in part because most data on HIV+HPgV co-infection comes from cross-sectional studies performed during the chronic phase of HIV infection. In particular, the impact of HPgV infection on early HIV infection–a period during which dramatic pathological changes in the HIV-infected host can shape the course of the infection [14]–has only been assessed using blood samples and with limited longitudinal sampling [8–10]. As such, the impact of HPgV co-infection on the natural course of HIV infection, and the mechanism(s) by which HPgV attenuates HIV disease *in vivo*, remain largely uncharacterized.

Macaque monkeys infected with simian immunodeficiency virus (SIV) exhibit several features of progressive HIV disease in humans, including CD4+ T cell depletion and pathological immune activation. As such, macaques infected with SIV are a valuable model for investigating the pathogenesis of HIV *in vivo* [15]. We recently discovered simian pegiviruses (SPgVs) infecting wild baboons in Africa [16] and used blood from an olive baboon (*Papio anubis*) sampled in Kibale, Uganda to infect captive cynomolgus macaques (*Macaca fascicularis*) with SPgV (referred to previously as SPgVkob, but here simply as SPgV). This resulted in the first laboratory-animal model of HPgV infection [17]. Notably, SPgV infection causes persistent, high-titer viremia in macaques without eliciting signs of disease, recapitulating several defining features of HPgV infection in humans.

Here, we used SPgV and SIV infection in Mauritian cynomolgus macaques to model HPgV and HIV co-infection in humans. We compared SIV disease parameters in four SPgV+SIV co-infected macaques to four macaques infected with SIV-only, with the hypothesis that SPgV would attenuate SIV pathogenesis during the acute phase of SIV infection, or result in improved recovery from acute insult of SIV infection, as is observed in African monkeys which are often co-infected with their own species-specific SIVs and SPgVs [18,19].

## Results

### SPgV pre-infection does not impact acute-phase SIV viremia

**Fig 1:**
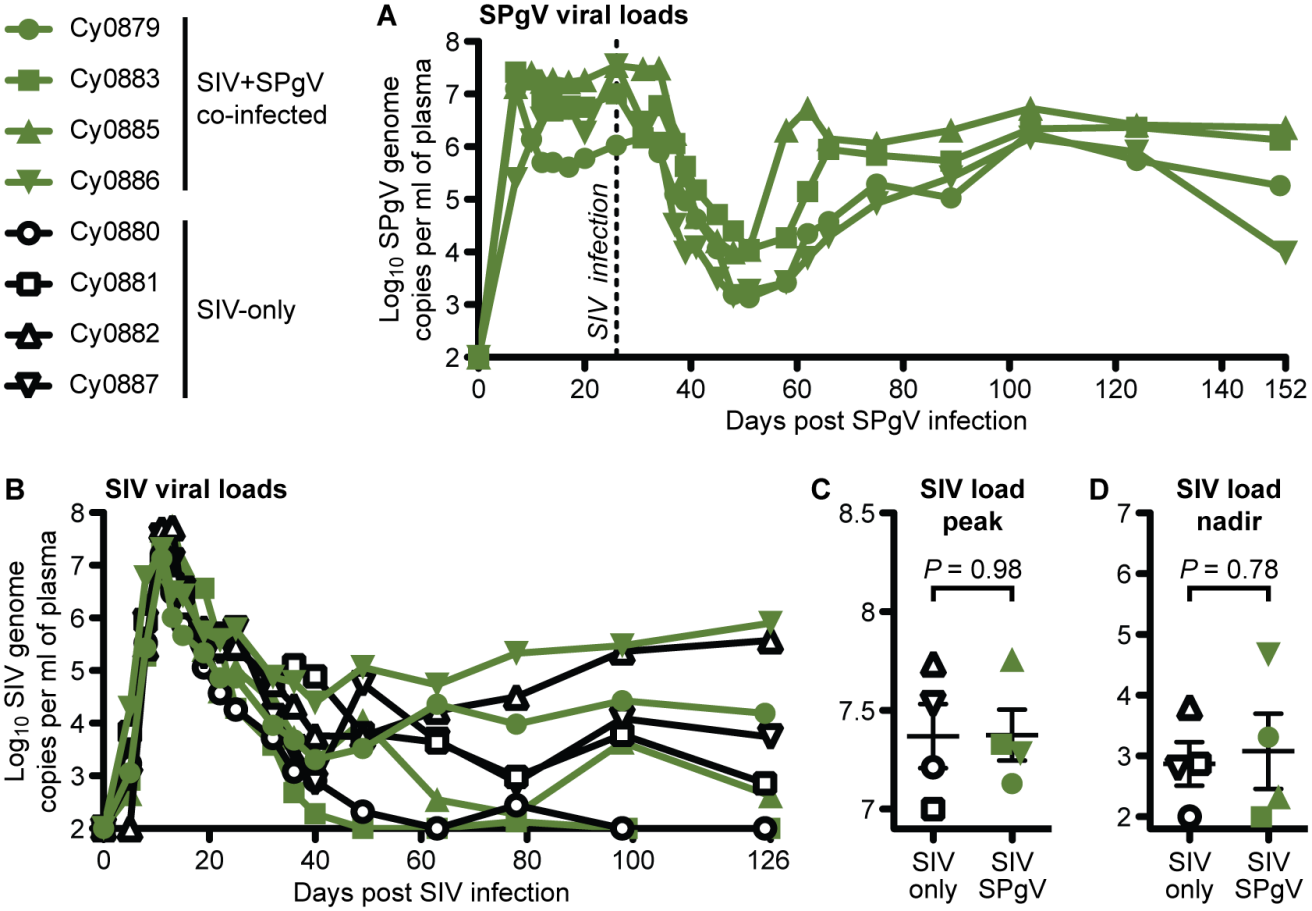
SPgV and SIV viral loads in infected macaques. Titers for each virus were measured from plasma using highly sensitive virus-specific quantitative RT-PCR assays. (**A**) SPgV titers in the four macaques infected with SPgV+SIV. (**B**,**C**,**D**) SIV titers in four macaques infected with SPgV+SIV (green) and four macaques infected with SIV-only (black). *P* values reflect a two-tailed unpaired t-test and error bars represent SEM. The symbols used for each animal in this figure are consistent throughout the manuscript.

For this study, eight female cynomolgus macaques (*Macaca fascicularis*) with identical major histocompatibility complex (MHC) class I haplotypes (M3/M4 heterozygous) were randomized to receive SPgV or no intervention prior to SIV infection (S1 Fig, S1 Table). For the experimental group, we chose to infect with SPgV prior to SIV infection so that we could observe the effect of established SPgV infection on SIV disease trajectory during acute SIV infection. Four macaques received intravenous inoculations of plasma (from cy0500 described in [17]) containing 2.29×10^7^ genome copies (gc) of SPgV as measured by quantitative RT-PCR. These infections achieved initial peak titers between 7.30×10^6^ and 2.66×10^7^ gc/ml of plasma between 7 and 14 days post SPgV infection (Fig 1A), similar to what had been observed previously. By 26 days post-SPgV infection viral loads had established a high-titer steady-state (average of 2.02×10^7^ gc/ml) and all eight macaques were inoculated intra-rectally with 7,000 tissue-culture-infectious-dose (TCID)_50_ of SIVmac239.

SIV plasma viral loads followed a typical trajectory during acute phase, reaching peak titers between 1.01×10^7^ and 5.25×10^7^ gc/ml of plasma between days 11 and 13 post-SIV infection in all eight macaques (Fig 1B). No differences in peak SIV plasma titer or post-peak nadir were observed between the SIV+SPgV co-infected and SIV-only groups (Fig 1C). To determine whether SPgV impacted subsequent SIV viral load trajectory, we followed macaques for 126 days after SIV infection. Within each group, we observed a wide range of viral load set-point titers. However, there was not a significant difference in SIV viral loads between the SIV-only and SIV+SPgV groups at any time point (Fig 1D).

### Acute SIV infection reduces SPgV viremia

Beginning as early as day 5 of SIV infection (day 31 of SPgV infection), we observed a significant drop in SPgV plasma viral loads in all SPgV+SIV co-infected macaques. The decline in SPgV viral loads reached a nadir of 1.36×10^3^–1.11×10^4^ gc/ml of plasma between day 22 and 25 of SIV infection (day 48–51 of SPgV infection), then gradually rebounded to a new set-point that was approximately 1.5 log_10_ lower than the pre-SIV infection set-point by day 40–49 of SIV infection (66–75 of SPgV infection). Previously, we showed that SPgV accumulates little-to-no sequence variation over time in infected macaques. Therefore, we deep sequenced the SPgV genome from each animal before the decline (day -6 of SIV infection; day 20 SPgV infection) and after recovery of high-titer SPgV viremia (day 49 of SIV infection; day 75 SPgV infection) to look for unique signatures of immune pressure on SPgV that may have been triggered by SIV infection. While SPgV from two macaques accumulated 1–3 protein-coding (*i*.*e*. non-synonymous) mutations over this period, SPgV from the other two macaques revealed no protein-coding mutations (S2 Table), suggesting that an SPgV-specific immune response and subsequent mutational escape was not responsible for the measured decrease in SPgV viral loads.

We hypothesized that the reduction in SPgV viral load during acute SIV infection was the result of inflammation induced by SIV, which has been reported to occur secondary to microbial translocation from the gut lumen [14]. Thus, we infected eight macaques intravenously with 2.29×10^7^ gc of SPgV and treated four of these macaques with dextran sulfate sodium (DSS) on day 26 post-SPgV infection to induce microbial translocation [20,21]. DSS treatment had no significant impact on SPgV viral loads, suggesting that inflammation caused by microbial translocation during acute SIV infection was not responsible for the observed decline in SPgV viral loads (S3 Table).

### SPgV pre-infection does not prevent loss of peripheral CD4+ T cells

**Fig 2:**
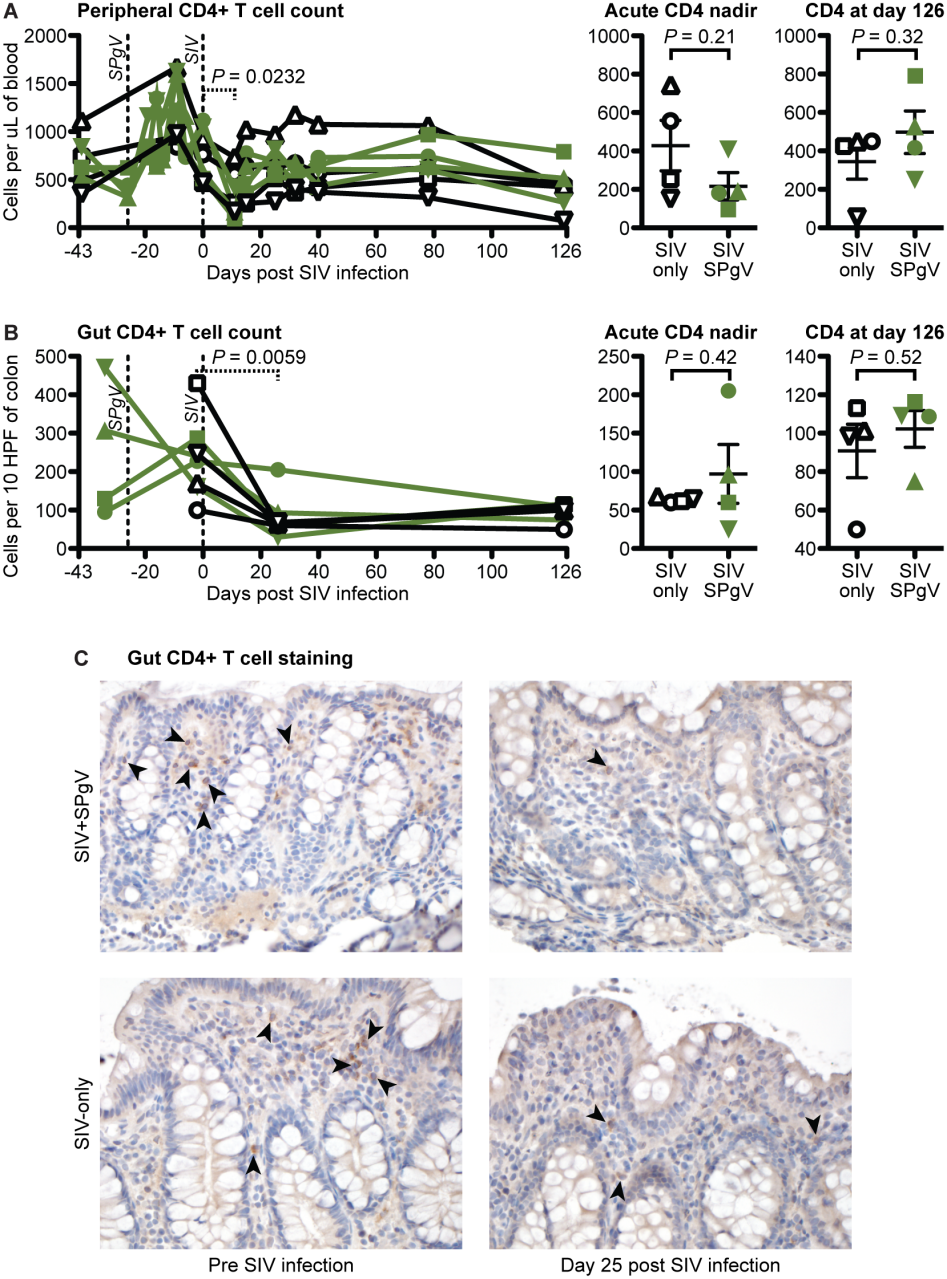
SIV pathogenesis in SIV-only vs. SIV+SPgV infected macaques. (**A**) Peripheral CD4+ T cell counts were obtained by multiplying absolute lymphocyte counts by the percentage of lymphocytes that were CD3+ CD4+ CD20- CD8- (see Fig 3 for gating strategy details). (**B**) Gut CD4+ T cells were stained within sections of colonic tissues via IHC with an anti-CD4 antibody and manually quantified. Significant differences between the SIV-only and SPgV+SIV groups were analyzed using a two-tailed unpaired t-test (solid line) with error bars representing SEM. Significant changes in all animals over the course of acute SIV infection were quantified using a two-tailed paired t-test (dashed line). (**C**) A representative set of colonic tissue from Cy0883 (SIV+SPgV) and Cy0887 (SIV-only) are shown pre and post SIV infection at 400x for comparison. Arrows highlight representative cells with membranous CD4 staining.

The absolute number of circulating CD4+ T cells remains the most clinically-relevant marker of HIV/SIV disease progression, and some studies of HIV+ human patients have shown a modest association between HPgV co-infection and higher peripheral CD4+ T cell counts [5–8]. Therefore, we followed blood CD4+ T cell counts in both the SIV-only and SIV+SPgV groups. We observed an initial drop in the circulating CD4+ T cells that corresponded with peak SIV viremia in all eight animals which then recovered to near pre-SIV levels. However, there were no statistically significant differences in the CD4+ T cell count between the SIV-only and SIV+SPgV groups (Fig 2A). An increase in the absolute number of circulating CD4+ T cells was observed prior to SIV infection in the macaques infected with SPgV, although a concomitant increase was also noted in the SPgV-naïve macaques during this time period, suggesting that this increase was not due to SPgV infection.

### SPgV pre-infection does not prevent loss of gut CD4+ T cells

The early loss of CD4+ T cells in the gastrointestinal tract is a hallmark of HIV/SIV pathogenesis [14,22]; yet the effect of HPgV/SPgV co-infection on gut CD4+ T cell depletion has never been examined. To see if SPgV pre-infection protected gut CD4+ T cells from SIV-mediated destruction, we collected colon pinch-biopsies from animals pre- and post-SIV infection, then analyzed the abundance of lamina propria CD4+ cells using immunohistochemistry (IHC). As expected, SIV infection led to an acute loss of gut-resident CD4+ cells, but the loss in the SIV+SPgV group was not statistically different compared to the SIV-only group (Fig 2B and 2C).

### SPgV pre-infection does not reduce pathological immune activation during acute SIV infection

**Fig 3:**
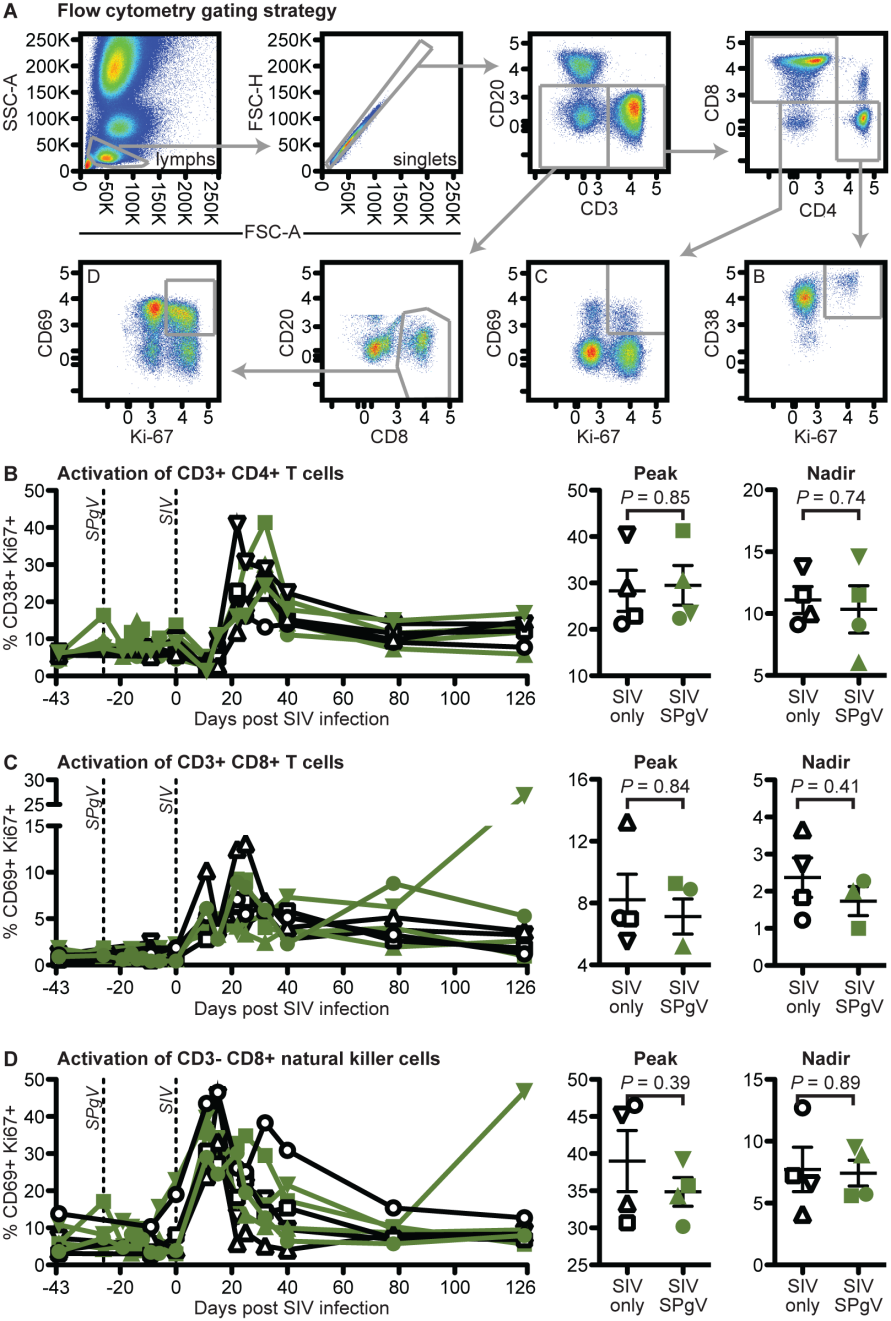
Peripheral immune activation in SIV-only vs. SIV+SPgV infected macaques. (**A**) Flow cytometry gating strategy used for defining immune cell subsets. Fresh whole blood was used for staining and flow cytometry at each time point. (**B-D**) Activation of immune cell subsets. *P* values represent a two-tailed unpaired t-test with error bars reflecting SEM. Note: Cy0886 did not exhibit a distinct peak or nadir of CD69+ Ki67+ expression in the CD3+ CD8+ T cell population, and so is not included in these analyses.

**Fig 4:**
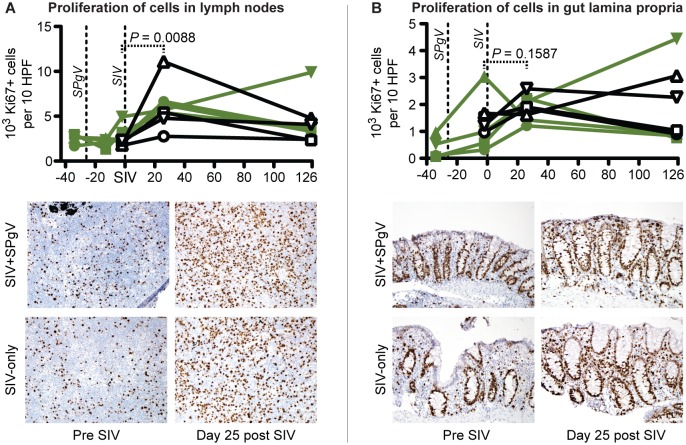
Activation of immune tissues in SIV-only vs. SIV+SPgV infected macaques. Proliferating cells were stained within sections of lymph nodes (**A**) and colon (**B**) via IHC with an anti-Ki67 antibody and manually quantified. Significant changes over time were quantified using a two-tailed paired t-test (dashed line). A representative set of lymph nodes from Cy0883 (SIV+SPgV) and Cy0881 (SIV-only) is shown at 400X pre and post SIV infection for comparison in (**A**). A representative set of colon tissues from Cy0886 (SIV+SPgV) and Cy0887 (SIV-only) is shown pre and post SIV infection at 400x for comparison in (**B**).

Several studies have demonstrated a correlation between HPgV infection and reduced immune activation in HIV-infected people [9–12]. However, none of these studies have examined the effect of HPgV pre-infection on the trajectory of HIV disease during acute HIV infection, a time when HIV is known to cause irreversible damage to the immune system. Therefore, we trended changes in peripheral immune cell activation after SIV infection using flow cytometry. For each immune cell subset examined (CD3+CD4+ T cells, CD3+CD8+ T cells, and CD3-CD8+ natural killer [NK] cells) we chose a combination of activation markers that most clearly delineated a positive and negative population (Fig 3A). We also used the proliferation marker Ki67 in our staining panel, as lymphocyte proliferation is indicative of a mounting immune response and proliferating CD4+ T cells are preferentially infected by SIV. While the timing and magnitude of activation following SIV infection varied by cell subset, we did not detect a significant difference in the magnitude of peak activation, the time to peak activation, or the post-peak nadir of activation between the SIV-only and SIV+SPgV groups within any subset (Fig 3B–3D). Activation of immune cells in the gut and in lymph nodes, as measured by IHC staining for the proliferation marker Ki67, showed a similar pattern (Fig 4).

### SPgV infection does not induce activation of the immune system

**Fig 5:**
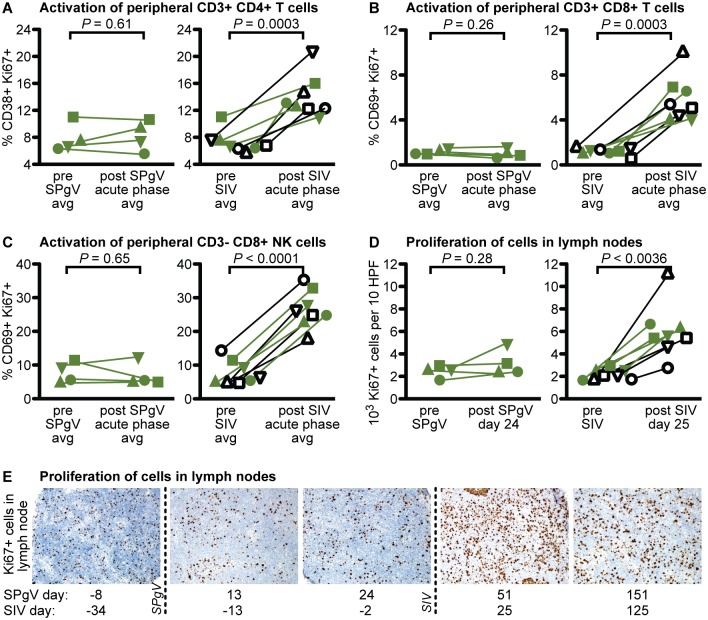
Immune activation following SPgV vs. SIV infection. (**A-C**) Fresh whole blood was used for analysis by flow cytometry at each time point. *P* values are from a two-tailed paired t-test comparing the average immune activation pre-any-virus-infection to the average of all post-SPgV or post-SIV data points within the first 26 days of infection for each virus for which flow cytometry data was available. (**D**) Proliferating cells were stained within sections of lymph nodes via IHC with an anti-Ki67 antibody and manually quantified (SPgV: day -8 vs day 24; SIV: day -34 vs day 25). Significant changes over time were quantified using a two-tailed paired t-test. (**E**) Representative set of lymph node tissue from Cy0885 is shown at 400x.

Systemic viral infections typically elicit a Th1-type immune response characterized by the activation of lymphocytes. To examine the immune response to acute pegivirus infection, we trended markers of immune cell activation by flow cytometry for 26 days following SPgV infection. Oddly, we saw no significant changes from pre-infection baseline in the total number or activation state of circulating lymphocytes during this time period despite high titers of SPgV. This is in stark contrast to SIV infection, which elicited a robust increase in immune activation during the same timeframe (Fig 5).

### SPgV infection does not elicit SPgV-specific antibodies, but does not alter production of SIV-specific antibodies

**Fig 6:**
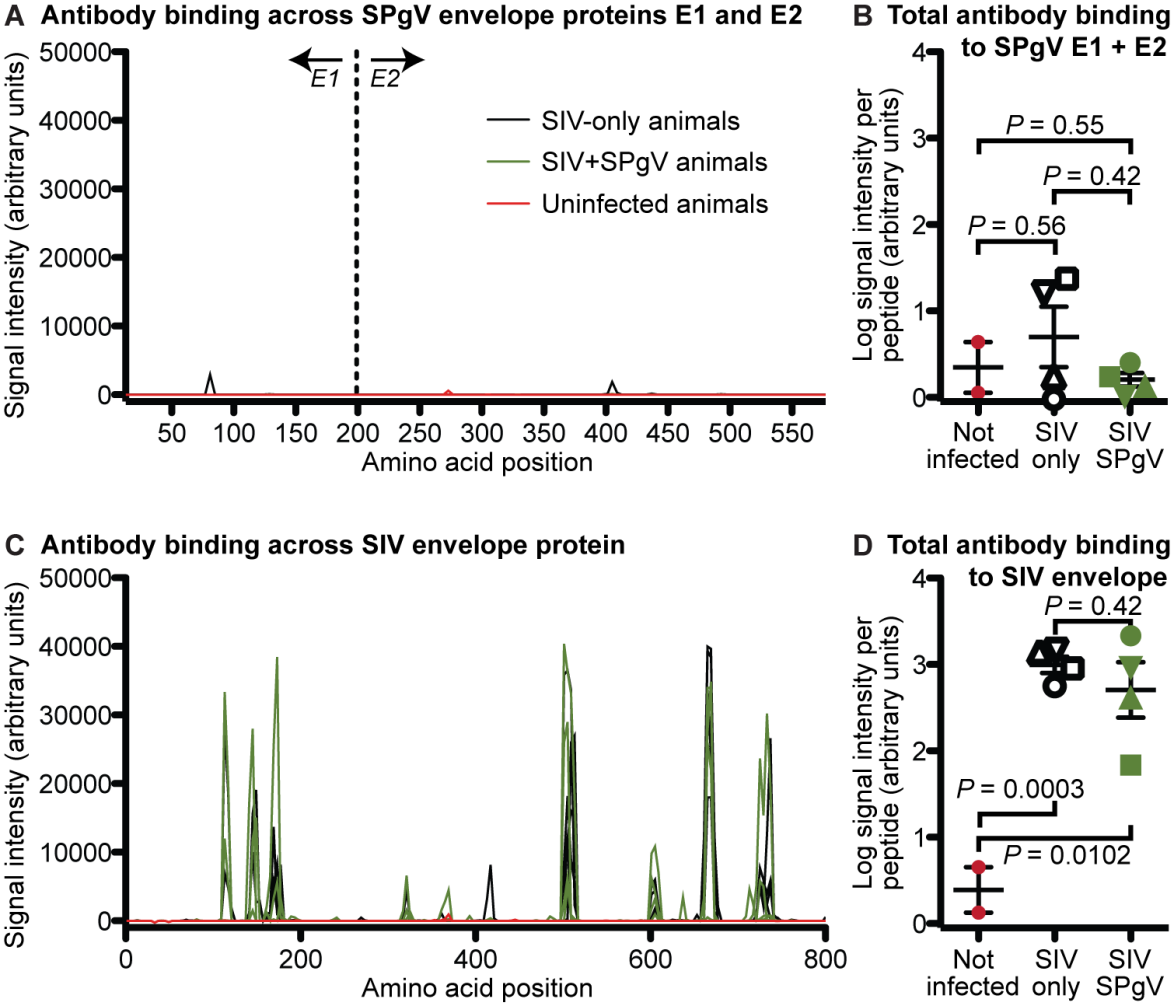
Antibody responses to linear epitopes in the SPgV and SIV envelope proteins. Amino acid sequences from the envelope proteins of the viruses used in this study (E1/E2 of SPgV and env of SIV) were represented as 12–16 amino acid length peptides. Overlapping peptides (step size of 2 amino acids with peptides overlapping by 10–14 amino acids) were then synthesized and tiled on an array. Plasma from each animal in the study, collected 125 or 126 days post SIV infection, was then incubated on the array and antibody binding to each peptide was quantified using a mouse anti-primate-IgG secondary antibody. Signal intensity for each peptide spanning SPgV E1/E2 (**A**) and SIV env (**C**) is shown for animals infected with SIV-only (black), SIV+SPgV (green), or uninfected controls. Total signal intensity, normalized to the average signal per peptide in the SPgV E1/E2 array (**B**) and the SIV env array (**D**), is shown for each animal in the study along with uninfected controls. *P* values represent a two-tailed unpaired t-test with error bars reflecting SEM.

Antibodies are associated with clearance of HPgV viremia [23–25] and can play an important role in the control of HIV infection [26]. To examine antibody responses to SPgV, and the effect that SPgV infection might have on antibody responses to SIV, we developed a peptide-based antibody capture assay that arrayed overlapping peptides spanning the entire SPgV and SIV envelope proteins, enabling us to examine antibody responses to linear epitopes for both viruses. Plasma from animals infected with SIV-only and SIV+SPgV, collected at day 125/126 post-SIV infection, showed no differences in antibody binding to the SPgV peptide array compared to plasma from uninfected controls or compared to each other (Fig 6A and 6B). In contrast, plasma from animals infected with both SIV-only and SIV+SPgV contained antibodies that bound to SIV peptides corresponding to specific regions within the SIV envelope protein (Fig 6C). Quantitatively, the signals for SIV-specific antibodies in the plasma of animals infected with SIV-only and SIV+SPgV were several orders of magnitude higher than the signals observed for plasma from uninfected controls, but were not significantly different from one another (Fig 6D).

### SPgV infection does not alter SIV-specific CD8+ T cell responses

**Fig 7:**
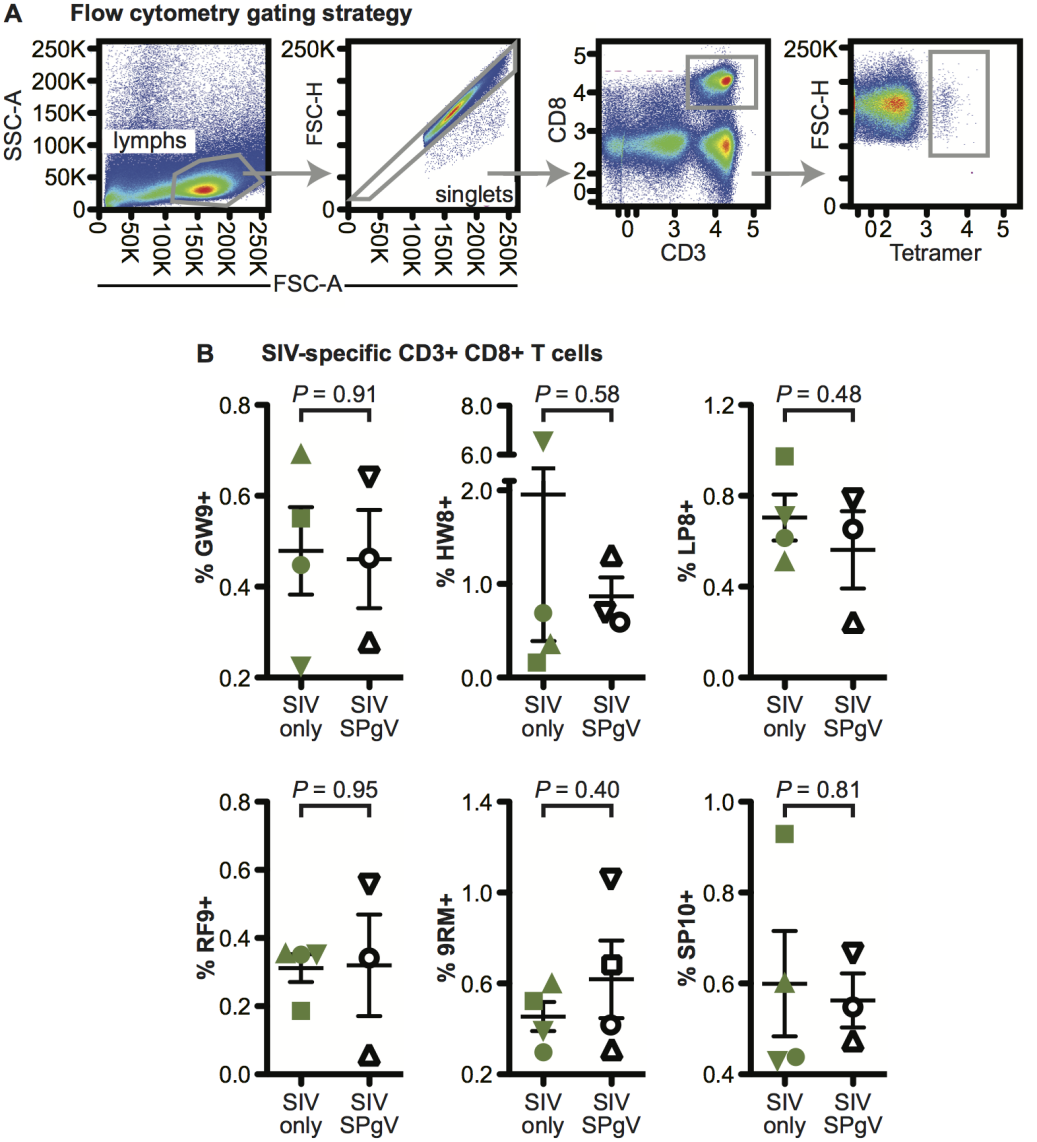
SPgV co-infection does not alter recognition of MHC class I restricted SIV epitopes by CD8+ T cells. Lymph nodes were collected from macaques at 125/126 days post SIV infection and cells were stained for analysis with MHC class I tetramers folded with SIV peptides that are immunodominant on the M3/M4 MHC background. (**A**) Flow cytometry gating strategy used for defining tetramer-positive CD8+ T cells. (**B**) Percentage of CD8+ T cells that were positive for each tetramer. *P* values represent a two-tailed unpaired t-test with error bars reflecting SEM.

CD8+ T cell responses are associated with the control of HIV/SIV infection in the context of certain MHC genetic backgrounds [27–29]. To evaluate the effect of SPgV infection on the development of SIV-specific CD8+ T cell responses, we stained lymph node-resident CD8+ cells purified from animals infected with SIV-only and SIV+SPgV at 125/126 days post-SIV infection using peptide-MHC class I tetramers, and examined the percentage of CD8+ cells that were also tetramer-positive. In all, we examined CD8+ T cell responses to six SIV epitopes that are immunodominant on the M3/M4 MHC background [30] (GW9, HW8, LP8, RF9, RM9, and SP10) but found no significant differences between the SIV-only and SPgV+SIV groups (Fig 7).

## Discussion

HIV+ people experience slower disease progression and reduced mortality when co-infected with HPgV, but the mechanisms by which HPgV mediates this protective effect are not known. Here, we utilized a recently-developed macaque model to study SPgV+SIV co-infection and found that pre-infection with SPgV had no effect on acute-phase SIV pathogenesis as measured by SIV viral loads, peripheral and gut-resident CD4+ T cell depletion, SIV-induced immune activation, or adaptive immune responses to SIV. One interpretation of these findings could be that, unlike HIV+HPgV co-infection in humans, SPgV does not protect macaques from SIV disease. While this is possible, studies of SPgV in non-human primates have shown that the biology of this virus closely mirrors HPgV in humans [1,16,17,19,31]. The progression of SIV infection in macaques also closely mirrors that of HIV infection in humans, and so it appears that SIV+SPgV infection in macaques is a close approximation of HIV+HPgV in humans. Another explanation for our findings could be that the group sizes in our study were considerably smaller than the group sizes in the human studies in which a protective effect of HPgV has been observed during HIV infection, thus rendering us unable to detect a small but significant protective effect. Nonetheless, extrapolation of our data to humans suggest that HPgV does not exert a major protective effect on HIV pathogenesis during the acute phase of HIV infection. Extending from this observation, we hypothesize that HPgV alters HIV/AIDS disease during the chronic phase, as some epidemiologic data on chronic HIV+HPgV co-infection also suggests [7,8,13,32]. Unfortunately, confirming and studying an effect on this timescale (*i*.*e*. years) is intractable in macaques. Nonetheless, a protective effect in the chronic phase of HIV infection may ultimately prove to be of greater clinical value, should a HPgV-inspired anti-HIV therapy ever come to fruition.

In addition to the observed protective effect of HPgV co-infection in HIV+ people, pegiviruses are able to evade the host immune system and maintain persistent infection via an unknown mechanism [17,31,33–36]. Thus, we studied immune responses to SPgV (and immune responses to SIV in SIV+SPgV co-infection animals) in detail. First, we examined broad markers of immune cell activation following SPgV and SIV infection in the blood, lymph nodes, and gut tissue. Following SPgV infection, we could not detect significant changes in immune activation for any cell type in any tissue despite high levels of virus replication. In contrast, SIV infection evoked significant increases in immune cell activation in all cell types in all tissues examined. Similarly, we examined antibody responses to linear SPgV epitopes in infected macaques using a custom-built antibody-capture array. We found no evidence for an antibody response, despite detecting robust antibody responses to linear SIV epitopes in the same animals using the same technology, which is consistent with previous studies showing a lack of anti-HPgV antibodies in individuals viremic with HPgV [8,25,37]. Together, these data are indicative of an absent immune response to SPgV and suggest that similar observations in HIV+HPgV co-infected humans is a true feature of HPgV biology and not due to immunosuppression by HIV [9,11,38,39]. In broader context, the lack of a host response to SPgV infection is striking: indeed, the few RNA viruses capable of maintaining high titers in mammalian hosts over long periods of time induce inflammation that disrupts tissue architecture, impairs immune system function, and ultimately exacerbates the disease-state of the host. While this process is perhaps most well described for HIV and SIV, inflammation and immunopathology play a central role in the biology of several other RNA viruses that persist in both primate and non-primate hosts [40–44]. Thus, the ability of SPgV to replicate without triggering a robust immune response also potentially provides a compelling explanation for its lack of pathogenicity. It remains to be determined whether pegiviruses inhibit activation of the immune system (leaving immune responses to SIV unaffected), or simply avoid immune recognition (despite sustaining high levels of viremia/antigenemia). Nonetheless, either scenario would require that these viruses employ a unique mechanism to maintain high-titer, persistent infection in the mammalian host.

Although pegivirus infection does not appear to elicit a substantial immune response, the significant drop in SPgV plasma viral loads that we observed upon co-infection of macaques with SIV suggests that pegivirus replication may be restricted by the immune activation which occurs as a result of acute SIV infection. One of the major drivers of immune activation during early SIV/HIV infection is the translocation of microbial products from the gut lumen into systemic circulation, which occurs as a result of the destruction of gut-resident CD4+ T cells. To determine whether this impacted SPgV replication, we treated SPgV infected macaques with DSS, a chemical known to induce microbial translocation. These macaques experienced no decrease in SPgV plasma viral loads, in contrast to SPgV+ monkeys infected with SIV. This possibly suggests that antiviral innate immune factors induced by SIV were responsible for decreased SPgV replication during the acute-phase of SIV infection. Alternatively, SPgV and SIV could share the same target cell-type, and the destruction of these cells by SIV could explain the temporary reduction in SPgV plasma viral loads. Indeed, an inverse correlation between HIV load and HPgV load has been noted [7,45]. Both viruses are lymphotropic, but SPgV and HPgV primarily exhibit bone-marrow tropism [17,46–48] while SIV and HIV target mature CD4+ T cells residing mostly in secondary lymphoid organs [49]. Defining the cell types infected by SPgV/HPgV and SIV/HIV is an active area of research, and recent studies may suggest that these viruses share a common target cell in the bone marrow [50] or in circulating lymphocytes [51]. However, the development of new *in vitro* tools will be essential for identifying the SPgV/HPgV cellular receptor(s) and interrogating the molecular determinants of SPgV/HPgV tropism. We are hopeful that future pegivirus studies will provide a deeper understanding of the biology of these enigmatic viruses, and ultimately the mechanisms by which HPgV protects humans from HIV-associated disease.

## Materials and methods

### Ethics statement

All macaque monkeys used in this study were cared for by the staff at the Wisconsin National Primate Research Center (WNPRC) following the guidelines outlined in the National Research Council Guide for the Care and Use of Laboratory Animals, the recommendations from the Association for Assessment and Accreditation of Laboratory Animal Care (AAALAC), and the Weatherall Report. Details of this study were approved by the University of Wisconsin Institutional Animal Care and Use Committee (permit G00707). Monkeys were group-housed until they were infected with SPgV or SIV, at which point they were singly housed to prevent spread of infections in the colony. Once singly housed, all animals had visual and auditory contact with each other in the same room. Monkeys were fed twice daily with commercial chow (Harlan Teklad #2050, 20% protein Primate Diet, Madison, WI) and given a variety of fruit in the afternoons. Foraging activities and physical environmental enrichment were also provided at least once weekly for both activities. Housing rooms were maintained at 65–75°F, 30–70% humidity, and on a 12:12 light-dark cycle (On: 0600, Off: 1800). All monkeys were evaluated for signs of pain, illness, and stress by observing appetite, stool, typical behavior, and physical condition, by the staff of the Animal Services Unit at least twice per day. WNPRC veterinary staff were notified upon identification of abnormalities and their recommendations were subsequently implemented. For euthanasia, animals were anesthetized with ketamine (at least 15 mg/kg IM) or other form of WNPRC veterinary approved general anesthesia followed by an IV overdose (greater than or equal to 50 mg/kg or to effect) of sodium pentobarbital or equivalent as approved by a WNPRC veterinarian.

### Selection of animals

To control for host genetic factors to the extent possible, we used cynomolgus macaques from the island of Mauritius, where there is an inbred macaque population due to a recent founder effect. All animals selected for this study were female and were major histocompatibility complex (MHC)-matched: all animals were heterozygous with an M3/M4 combination of MHC haplotypes. Unlike other defined MHC haplotypes found in Mauritian cynomolgus macaques (*e*.*g*. M1), spontaneous control of SIV infection is not known to be associated with the M3 or M4 haplotype [52].

### Virus inoculations

A SPgV stock was created for this study by aliquoting plasma collected from a macaque (cy0500) inoculated intravenously with plasma from an SPgV+ olive baboon (*Papio anubis*) sampled in Kibale National Park, Uganda (GenBank accession: KF234530), as described in detail previously [16,17]. Macaques infected with SPgV in this study were inoculated intravenously with 700 μL of cy0500 plasma containing 2.29 × 10^7^ genome copies of SPgV. SIV infections were achieved using a single intrarectal inoculation of 7,000 tissue-culture-infectious-dose (TCID)_50_ of the molecularly cloned SIVmac239 virus (GenBank accession: M33262).

### Chemical induction of microbial translocation

Microbial translocation was induced as described previously [21]. Briefly, a 0.5% solution of dextran sulfate sodium (DSS) was prepared by resuspending colitis-grade DSS (MPBio, Santa Ana, CA) in sterile drinking water and stored at 4°C. Animals were treated once per day for 5-consecutive days with 200 mL of the DSS-containing drinking water, administered by gavage. Animals were monitored for clinical signs of colitis and gastrointestinal distress, and received palliative and clinical care at the full discretion of WNPRC veterinarians.

### RNA extraction from plasma for sequencing and viral loads

RNA was extracted from 300 μL of EDTA-treated plasma using the Viral Total Nucleic Acid Purification Kit (Promega, Madison, WI) on a Maxwell 16 MDx instrument and eluted in 50 μL of DNAse/RNAse free water.

### SPgV viral loads

A Taqman quantitative RT-PCR (qRT-PCR) assay was used to quantify viral RNA for SPgV (forward primer: 5’-CGGTGTTCATGGCAGGTAT-3’; reverse primer: 5’-CAGTTACAGCCGCGTGTTT-3’; probe: 5’-6FAM-ATGCACCCTGATGTAAGCTGGGCAA-BHQ1-3’), as described previously [16]. Reverse transcription and PCR were performed using the SuperScript III One-Step qRT-PCR system (Invitrogen, Carlsbad, CA) on a LightCycler 480 instrument (Roche, Indianapolis, IN). Reverse transcription was carried out at 37°C for 15 minutes and then 50°C for 30 minutes followed by 2 minutes at 95°C, and then 50 cycles of amplification as follows: 95°C for 15 sec and 60°C for 1 minute. The 20 μL reaction mixture contained 5 μL of extracted RNA, MgSO_4_ at a final concentration of 3.0 mM, with the two amplification primers at a concentration of 500 nM and probe at a concentration of 100 nM. RNA copy number was calculated using a standard curve that was sensitive down to 10 copies of RNA transcript per reaction.

### SIV viral loads

A Taqman qRT-PCR assay was used to quantify viral RNA for SIV (forward primer: 5′-GTCTGCGTCATCTGGTGCATTC-3′; reverse primer: 5′-CACTAGCTGTCTCTGCACTATGTGTTTTG-3′; probe: 5’-6FAM-CTTCCTCAGTGTGTTTCACTTTCTCTTCTGCG-BHQ1-3′), as described previously [53]. Cycling conditions were: 37°C for 15 min, 50°C for 30 min, and 95°C for 2 min, followed by 50 amplification cycles of 95°C for 15 sec and 62°C for 1 min with ramp times set to 3°C/sec. The final reaction mixtures (20 μL total volume) contained 0.2 mM dNTPs, 3.5 mM MgSO4, 150 ng random hexamer primers (Promega, Madison, WI), 0.8 μL SuperScript III One-Step qRT-PCR enzyme mix, 600 nM of each amplification primer and 100 nM of probe.

### Amplicon-based sequencing of SPgV

SPgV was amplified with the Qiagen OneStep RT-PCR kit and five overlapping ~2.5kb amplicons. Primers were designed using Primer3 [54] in Geneious R9 (Biomatters, Auckland, NZ) (S4 Table). Cycling conditions were: 50°C for 30 minutes and 94°C for 2 minutes, followed by 40 cycles of 94°C for 15 seconds, 55°C for 30 seconds, and 68°C for 2.5 minutes, followed by a final extension at 68°C for 5 minutes. Amplicons were fragmented and sequencing adaptors were added using the Nextera DNA Sample Preparation Kit (Illumina, San Diego, CA). Deep sequencing was performed on the Illumina MiSeq, and sequence data were analyzed using Geneious Pro R9 (Biomatters, Auckland, NZ). Low quality (<Q40, Phred quality score) and short reads [<100 base pairs (bp)] were removed, and coding-complete genome sequences for SPgV were acquired by mapping reads to the reference sequence for the SPgVkob inoculum (Genbank ID KF234530) using the Geneious alignment tool at medium-low sensitivity. Consensus SPgV sequences from each animal at each time point were compared to the inoculum using a ClustalW alignment with an IUB cost matrix, gap open cost of 15, and gap extend cost of 6.66.

### Immune cell activation and CD4+ T cell counts

Staining for flow cytometry was performed on EDTA-anticoagulated whole blood as described previously (Pomplun, 2015). Briefly, 0.1 mL of EDTA-anticoagulated whole-blood was incubated for 15 min at room temperature in the presence of a mastermix of antibodies against CD38 (clone AT1, FITC conjugate, 20 μl), CD69 (clone TP1.55.3, ECD conjugate, 10 μL), CD3 (clone SP34-2, Alexa Fluor 700 conjugate, 3 μl), CD25 (clone M-A251, Brilliant Violet 421 conjugate, 5 μl), CD8 (clone SK1, Brilliant Violet 510 conjugate, 2.5 μl), CD20 (clone 2H7, Brilliant Violet 650 conjugate, 2 μl), CD4 (clone L200, Brilliant Violet 711 conjugate, 5 μl) antigens. All antibodies were obtained from BD BioSciences (San Jose, CA, USA), except the CD69-specific antibody, which was purchased from Beckman Coulter (Brea, CA, USA) and the CD38-specific antibody, which was purchased from Stemcell Technologies (Vancouver, BC, Canada). Cells were also stained with LIVE/DEAD Fixable Near-IR during this time (Invitrogen, Carlsbad, CA). Red blood cells were lysed using BD Pharm Lyse, after which they were washed twice in media and fixed with 0.125 mL of 2% paraformaldehyde for 20 min. After an additional wash the cells were permeabilized using Bulk Permeabilization Reagent from Invitrogen (Carlsbad, CA, USA). The cells were stained for 15 min with an antibody against Ki67 (clone B56, Alexa Fluor 647 conjugate, 5 μL) while the permeabilizer was present. The cells were then washed twice and resuspended in 0.125 mL of 2% paraformaldehyde for 20 min. After a final wash and resuspension with 125 μL PBS supplemented with 10% fetal bovine serum (fluorescence-activated cell sorting [FACS] buffer), all samples were run on a BD LSRII Flow Cytometer within 24 hrs. Flow data were analysed using Flowjo version 9.8.2. Absolute CD4+ T cell counts were determined by multiplying the absolute number of lymphocytes obtained by complete blood count by the percentage of lymphocytes that stained positive for CD3 and CD4 and negative for CD20 and CD8 by flow cytometry.

### Gut and lymph node histology

Tissues were collected from anesthetized macaques, fixed in 10% formalin, then embedded in paraffin (FFPE). Five micron thick sections were cut from FFPE blocks and mounted on charged slides. To remove paraffin, slides were baked at 80°C, treated with xylene (5 min x 3), and hydrated through graded alcohols to deionized water. Heat-induced epitope retrieval was performed in pH 9.0 Tris-EDTA solution (10mM tris base, 1 mM EDTA, 0.05% tween-20) for 3 minutes (Ki67) or 1 minute (CD4) in a Decloaking Chamber (Biocare Medical, Concord, CA). Slides were then rinsed with PBS and blocked with 0.3% H_2_O_2_ in PBS for 10 min at room temperature followed by serum [10% goat serum (Sigma, St. Louis, MO) in PBS] for 1 hr at room temperature. Slides were incubated with primary antibody in PBS with 1% goat serum and 0.1% Triton X-100 overnight at 4°C in dilutions of 1:800 for anti-Ki67 (BD Pharmingen, Franklin Lakes, NJ: 556003) or 1:100 for anti-CD4 (Leica, Weltzlar, Germany: NCL-L-CD4-368). Slides were then rinsed thrice with PBS and treated with Signal Stain Boost IHC Detection Reagent (HRP, Mouse) (Cell Signaling Technology, Beverly, MA) for 30 min at room temperature. Slides were then rinsed three times with PBS and treated with DAB substrate (Cell Signaling Technology, Beverly, MA) for 1 min and Mayer’s hematoxylin (Sigma, St. Louis, MO) for 1 min. Slides were then rinsed in H_2_O and dehydrated through graded alcohols to xylene.

Immunohistochemical (IHC) stains for Ki67 and CD4 were scored by a pathologist who was blinded to study design and treatment assignment. Quantification was reported as the average of 10 high-power fields (600X). In the lymph nodes, Ki67 positive cells were counted within the parafollicular region only, since germinal centers were universally positive. In the colon both Ki67 and CD4 were quantified within the lamina propria and crypt epithelial cells were excluded.

### Antibody response quantitation using peptide array

Complete sequences of SIVmac239 envelope and SPgV E1 and E2 were submitted to Roche Sequencing Solutions (Madison, WI) as part of an early access program for development of a high density peptide array. Overlapping, 12–16 amino acid length peptides were tiled at a 2 amino acid interval (10–14 amino acid overlap). The peptide sequences were synthesized *in situ* with a Roche Sequencing Solutions Maskless Array Synthesizer (MAS) by light-directed solid-phase peptide synthesis using an amino-functionalized plastic support (Greiner Bio-One, Kremsmünster, Austria) coupled with a 6-aminohexanoic acid linker and amino acid derivatives carrying a photosensitive 2-(2-nitrophenyl) propyloxycarbonyl (NPPOC) protection group (Orgentis Chemicals, Gatersleben, Germany). All macaque plasma samples collected at day 125/126 post SIV infection were analyzed on the same run. Samples were diluted 1:100 in binding buffer (0.01M Tris-Cl, pH 7.4, 1% alkali-soluble casein, 0.05% Tween-20). Diluted sample aliquots and binding buffer-only negative controls were bound to arrays overnight at 4°C. After binding, the arrays were washed 3× in wash buffer (1× TBS, 0.05% Tween-20), 10 min per wash. Primary sample binding was detected via 8F1-biotin mouse anti-primate IgG (NIH nonhuman primate reagent resource) secondary antibody. The secondary antibody was diluted 1:10,000 (final concentration 0.1 ng/μl) in secondary binding buffer (1x TBS, 1% alkali-soluble casein, 0.05% Tween-20) and incubated with arrays for 3 hours at room temperature, then washed 3× in wash buffer (10 min per wash) and 30 sec in reagent-grade water. The secondary antibody was labeled with Cy5-Streptavidin (GE Healthcare; 5 ng/μl in 0.5x TBS, 1% alkali-soluble casein, 0.05% Tween-20) for 1 hour at room temperature, then the array was washed 2× for 1 min in 1× TBS, and washed once for 30 sec in reagent-grade water. Fluorescent signal of the secondary antibody was detected by scanning at 635 nm at 2 μm resolution and 25% gain, using an MS200 microarray scanner (Roche NimbleGen, Madison, WI). Peptide signal intensities were processed by two correction factors. First, a statistical method was used to estimate background signal for an array by implementing a RMA background correction (performed in R), and this value was subtracted from the signal intensities. Next, a spatial correction was performed to correct for signal bias in the array associated with spatial bias by implementing a 2d loess correction (performed in R). Finally, the signal intensity of each peptide incubated without plasma was subtracted from the signal intensity generated for each plasma sample for the corresponding peptide.

### SIV-specific CD8+ T cell responses

Biotinylated peptide-MHC class I monomers (pMHCl) were provided by Dr. David Price and synthesized as described previously [55]. Tetramers were produced by mixing pMHCl monomers at a 4:1 molar ratio with purified streptavidin conjugated to phycoerythrin (PE). The tetramer staining protocol was modified slightly from a protocol described previously [56]. Briefly, ∼1 × 10^6 cells were re-suspended in 100 μl of R10 medium with 1 μg/ml of the appropriate tetramer for 30 min at 37°C. Cells were then surface stained with anti-CD3-AlexaFluor700 (clone SP34-2) and anti-CD8-BV510 (clone SK1) for 30 min at room temperature; washed with 1× PBS supplemented with 10% fetal bovine serum (fluorescence-activated cell sorting [FACS] buffer); and fixed with 2% paraformaldehyde (PFA) (Thermo Fisher Scientific, Waltham, MA). Samples were acquired by using an LSRII flow cytometer (BD Biosciences, Franklin Lakes, NJ), and data were analyzed with FlowJo software (TreeStar Inc., Ashland, OR).

### Statistical analysis

Information on statistical tests used to determine significance can be found in corresponding figure legends. All statistical analyses were performed in Graphpad Prism software version 6.0h (GraphPad Software, Inc., La Jolla, CA).

## Supporting information

S1 Fig Timeline of study. (TIFF)

S1 Table Sampling schedule and accompanying data. (XLSX)

S2 Table Sequencing statistics. (XLSX)

S3 Table DSS study details. (XLSX)

S4 Table Primers for SPgV sequencing. (XLSX)
