# Supplementary figures and images for: Pegivirus avoids immune recognition but does not attenuate acute-phase disease in a macaque model of HIV infection
Source: PLoS Pathog. 2017 Oct 26;13(10):e1006692. doi: 10.1371/journal.ppat.1006692 (PMC5675458; doi:10.1371/journal.ppat.1006692)

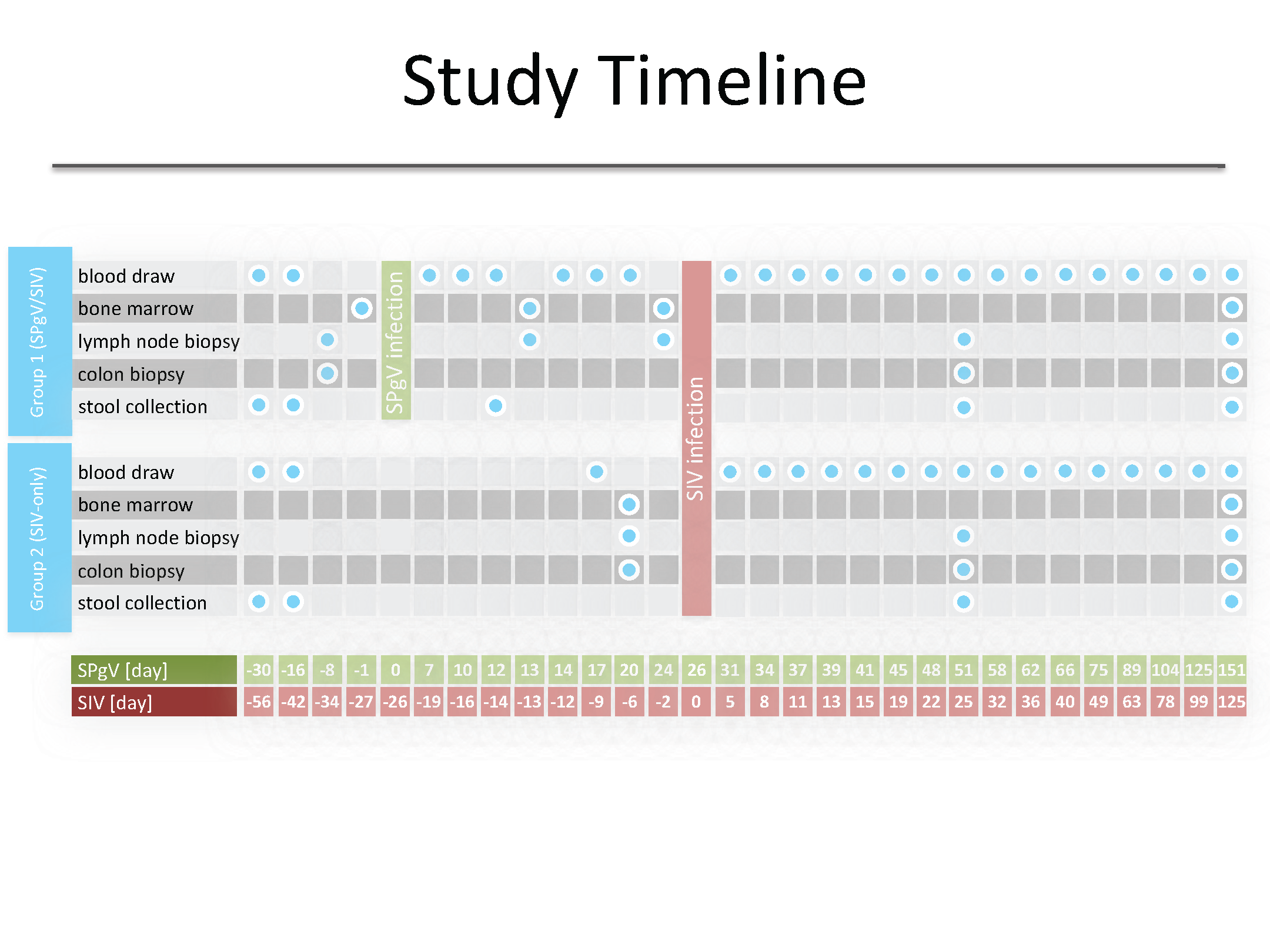

Supplement: S1 Fig — (TIFF) [file ppat.1006692.s001.tiff]
